# Mitral valve stenosis and left ventricular hemodynamic alterations after mitral valve repair

**DOI:** 10.1186/1532-429X-16-S1-O70

**Published:** 2014-01-16

**Authors:** Donald Zhang, Jeremy R McGarvey, Madonna Lee, Satoshi Takebayashi, Chikashi Aoki, Christen Dillard, Francisco Contijoch, Gerald Zsido, Qiao Han, Yuchi Han, James J Pilla, Joseph H Gorman, Walter R Witschey, Robert C Gorman

**Affiliations:** 1 Surgery, University of Pennsylvania, Philadelphia, Pennsylvania, USA

## Background

Mitral valve repair (MVR) shows clinical and economic superiority over mitral valve replacement in patients with dystrophic and rheumatic regurgitation, but outcomes are less certain in chronic ischemic mitral regurgitation (IMR). We hypothesize that MVR may have adverse effects on LV hemodynamics resulting from suboptimal annuloplasty geometry and post-repair mitral valve stenosis whose consequences are most acutely felt in IMR patients because of their impaired LV function. To test this hypothesis, we qualitatively described post-repair hemodynamics and measured LV inflow quantities (peak diastolic inflow velocity, transmitral pressure, and inflow angle) after MVR in sheep.

## Methods

The University of Pennsylvania IACUC approved all experiments. Dorsett swine (n = 7) were anesthetized, underwent baseline MRI and echocardiography followed by MVR with varying annuloplasty size and shape (24, 26, 30 and 32 mm Carpentier-Edwards Physio; Edwards Lifesciences, 30 mm Duran Ancore; Medtronic, and 26 or 30 mm Saddle (Profile 3D; Medtronic). At 1 week post-repair, all animals underwent a follow-up MRI and echocardiography. The 4D PC MRI data was post-processed to remove static tissues, residual aliasing, and eddy currents. Blood flow velocity vectors were visualized using stream- and pathlines (CEI Ensight) and hemodynamics were qualitatively summarized. Peak transmitral flow velocity, pressure (modified Bernoulli equation) and the angle of the inflow jet were computed at the mitral valve vena contracta.

## Results

**Figure 1:**
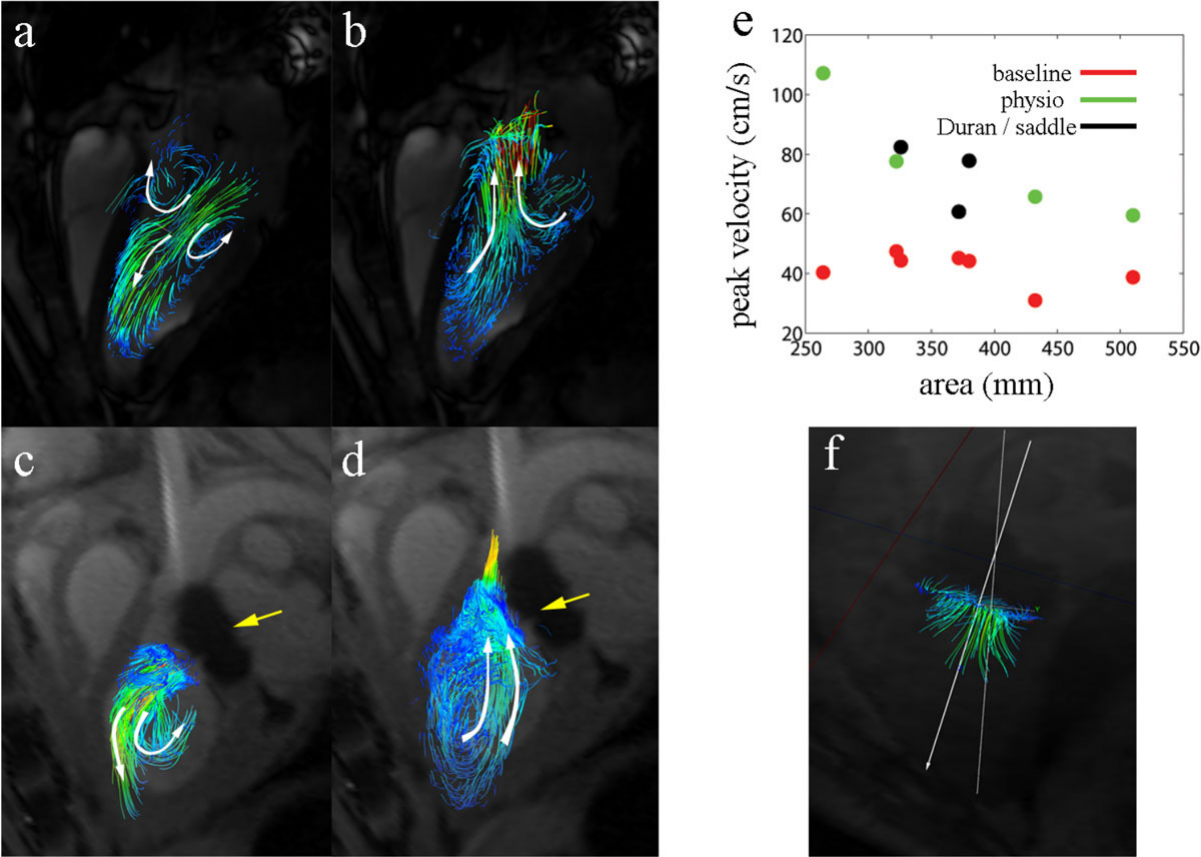
**Hemodynamics pre- and post-mitral valve repair in sheep**. White arrows indicate the direction of flow, yellow arrows indicate the repair ring artifact (not present in Duran rings) (a) baseline diastolic flow is oriented towards apex with two counter-rotating vortices. This is a single toroid in a 3D view (b) baseline systolic flow (c) 1 wk post-repair with a 24 mm Physio annuloplasty ring. The anterior vortex is dissipated and there is a single, large posterior vortex (d) 1 wk post-repair systolic flow in which posterior vortex continues to circulate and eject (e) correlation between post-repair peak velocity and annuloplasty ring area (f) inflow angle is difference from valve coaptation to apex and angle of flow directed towards vena contracta.

Diastolic transmitral flow in naïve sheep was characterized by a single intraventricular vortex (toroid) visualized by two counter-rotating vortices in the 3 chamber long-axis view. The posterior mitral valve vortex was slightly smaller than the anterior vortex. The transmitral pressure significantly increased (4 +- 0.2 mmHg, P < 0.05) in all animals post-repair causing mitral valve stenosis, including in the largest 32 mm Physio Ring. We found that there was a rotation 11.5 ± 1.3° to 19.8 ± 5.7° directed towards the anterior MV leaflet between the normal and post-repair diastolic inflow axis (n = 7, P < 0.1) as shown in Figure 1f. Smaller undersized rings had the most adverse affect on diastolic flow, resulting in a 25.7 and 30.5° rotation in 24 mm Physio and 26 mm Saddle shaped plasty, respectively. Torque on the mitral valve annulus caused by undersized rings could have caused clockwise, anteroapical rotation of the valvular apparatus, resulting in redirection of the flow jet towards the septal wall and disappearance of annular vortices. There was an inverse relationship between peak diastolic velocity and plasty area; a likely consequence of Bernoulli's law. Overall, peak MV velocity increased from 39.4 ± 6.7 to 77.5 ± 21.2 post-repair (n = 7, P < 0.05).

## Conclusions

Mitral valve repair can result in significant mitral valve stenosis and abnormal LV hemodynamics.

## Funding

The authors gratefully acknowledge support from the National Institutes of Health through awards K99HL108157 and R01HL63904.

